# (*E*)-*N*′-(4-Bromo­benzyl­idene)-*p*-toluene­sulfonohydrazide

**DOI:** 10.1107/S1600536809009751

**Published:** 2009-03-25

**Authors:** Reza Kia, Bijan Etemadi, Hoong-Kun Fun, Hadi Kargar

**Affiliations:** a X-ray Crystallography Unit, School of Physics, Universiti Sains Malaysia, 11800 USM, Penang, Malaysia; b Department of Earth Sciences, College of Sciences, Shiraz University, Shiraz, Iran; c Department of Chemistry, School of Science, Payame Noor University (PNU), Ardakan, Yazd, Iran

## Abstract

In the title compound, C_14_H_13_BrN_2_O_2_S, a novel sulfonamide derivative, inter­molecular N—H⋯O and C—H⋯O hydrogen bonds link neighbouring mol­ecules into different dimers along the *b* axis, generating *R*_2_^2^(8) and *R*_2_^2^(16) ring motifs. The dihedral angle between the benzene rings is 82.39 (13)°. The crystal structure is further stabilized by inter­molecular π–π stacking inter­actions [centroid–centroid distances = 3.867 (2)–3.9548 (8) Å].

## Related literature

For bond-length data, see: Allen *et al.* (1987 ▶). For hydrogen-bond motifs, see: Bernstein *et al.* (1995 ▶). For related structures and applications, see, for example: Kia *et al.* (2008*a* ▶,*b* ▶); Tabatabaee *et al.* (2007 ▶); Ali *et al.* (2007 ▶); Tierney *et al.* 2006 ▶; Krygowski *et al.* (1998 ▶); Mehrabi *et al.* (2008 ▶); Kayser *et al.* (2004 ▶). For the stability of the temperature controller used for the data collection, see: Cosier & Glazer (1986 ▶). 
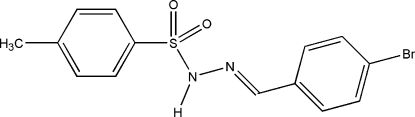


## Experimental

### 

#### Crystal data

C_14_H_13_BrN_2_O_2_S
*M* *_r_* = 353.23
Triclinic, 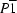

*a* = 5.9565 (3) Å
*b* = 9.4005 (3) Å
*c* = 12.8020 (6) Å
α = 97.153 (2)°
β = 96.350 (2)°
γ = 92.125 (1)°
*V* = 705.95 (5) Å^3^
*Z* = 2
Mo *K*α radiation
μ = 3.06 mm^−1^
*T* = 100 K
0.57 × 0.15 × 0.07 mm

#### Data collection

Bruker SMART APEXII CCD area-detector diffractometer
Absorption correction: multi-scan (**SADABS**; Bruker, 2005 ▶) *T*_min_ = 0.276, *T*_max_ = 0.819
18030 measured reflections
5040 independent reflections
4098 reflections with *I* > 2˘*I*)
*R*_int_ = 0.034

#### Refinement

*R*[*F* ^2^ > 2σ(*F* ^2^)] = 0.040
*wR*(*F* ^2^) = 0.106
*S* = 1.11
5040 reflections
187 parameters
H atoms treated by a mixture of independent and constrained refinement
Δρ_max_ = 0.63 e Å^−3^
Δρ_min_ = −0.56 e Å^−3^

### 

Data collection: *APEX2* (Bruker, 2005 ▶); cell refinement: *SAINT* (Bruker, 2005 ▶); data reduction: *SAINT*; program(s) used to solve structure: *SHELXTL* (Sheldrick, 2008 ▶); program(s) used to refine structure: *SHELXTL*; molecular graphics: *SHELXTL*; software used to prepare material for publication: *SHELXTL* and *PLATON* (Spek, 2009 ▶).

## Supplementary Material

Crystal structure: contains datablocks global, I. DOI: 10.1107/S1600536809009751/rz2299sup1.cif

Structure factors: contains datablocks I. DOI: 10.1107/S1600536809009751/rz2299Isup2.hkl

Additional supplementary materials:  crystallographic information; 3D view; checkCIF report

## Figures and Tables

**Table 1:** Hydrogen-bond geometry (Å, °)

*D*—H⋯*A*	*D*—H	H⋯*A*	*D*⋯*A*	*D*—H⋯*A*
N1—H1*N*1⋯O1^i^	0.85 (4)	2.06 (4)	2.902 (3)	171 (4)
C14—H14*A*⋯O1^ii^	0.98	2.57	3.420 (3)	145

Symmetry codes: (i) 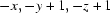; (ii) 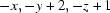.

**Fig. 1.**
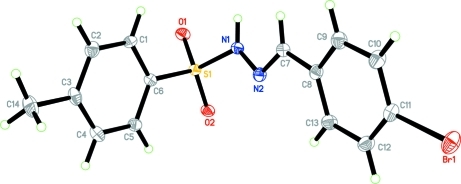
The molecular structure of the title compound, showing 50% probability displacement ellipsoids and the atomic numbering.

**Fig. 2.**
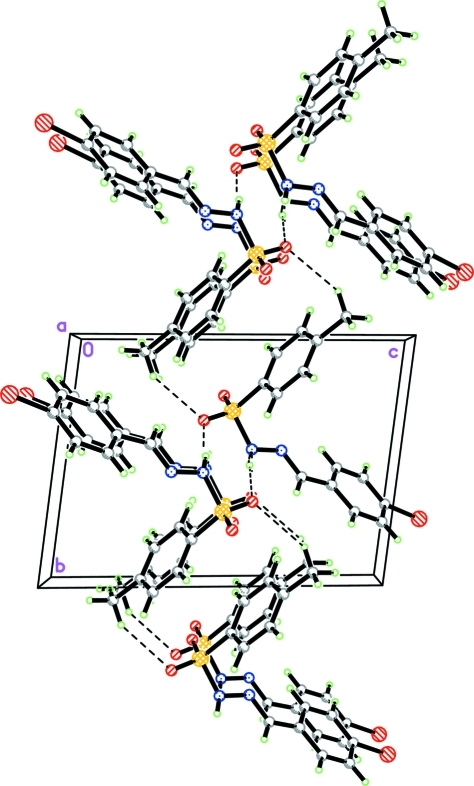
The crystal packing of the title compound, viewed down the a axis, showing linking of different dimers along the b axis through N—H···O and C—H···O interactions. Intermolecular hydrogen bonds are shown as dashed lines.

## supplementary crystallographic information

### Comment

Sulfonamides were the first class of antimicrobial agents to be discovered. They inhibit dihydropteroate synthetase in the bacterial folic acid pathway. Although their clinical role has diminished, they are still useful in certain situations, because of its efficacy and low cost (Krygowski *et al.*, 1998). Sulfonamides (sulfanilamide, sulfamethoxazole, sulfafurazole) are structural analogues of *p*-aminobenzoic acid (PABA) and compete with PABA to block its conversion to dihydrofolic acid. These agents are generally used in combination with other drugs (usually sulfonamides) to prevent or treat a number of bacterial and parasitic infections (Tierney *et al.*, 2006). Because of the above impotrtant features, we report the crystal structure of the title compound.

The title compund (Fig. 1), is a novel sulfonamide derivative. Bond lengths (Allen *et al.*, 1987) and angles are within the normal ranges and are comparable with the related staructures (Kia *et al.* 2008*a*,*b*; Mehrabi *et al.*, 2008; Ali *et al.* 2007). Intermolecular N—H···O and C—H···O hydrogen bonds link neighbouring molecules by *R*_2_^2^(8) and *R*_2_^2^(16) ring motifs (Bernstein *et al.*, 1995), respectively, into different dimers along the *b* axis. The dihedral angle between the two benzene rings is 82.39 (13)°. The crystal structure is further stabilized by intermolecular π-π stacking interactions [*Cg1*···*Cg1*^iii^ = 3.9319 (15) and *Cg2*···*Cg2*^iv^ = 3.8677 (16) Å; the perpendicular distances are 3.6548 (10) and 3.6189 (11) Å, respectively. Symmetry codes: (iii) -*x*, 2 - *y*, 1 - *z*; (iv) 2 - *x*, 1 - *y*, 2 - *z*].

### Experimental

*p*-Tosylhydrazine (2 mmol) was added to a 50 ml refluxing ethanolic solution of 4-bromobenzaldehyde (2 mmol). The mixture was stirred for 2 h. After cooling, the colorless crystalline solid was isolated by filtration, washed with cold ethanol, and re-crystallized from ethanol.

### Refinement

H atom bound to N1 was located from the difference Fourier map and refined freely; see Table 1. The rest of the hydrogen atoms were positioned geometrically and refined as riding model. A rotating group model was used for the methyl group. The highest peak (0.63 e.Å^-3^) is located 1.77 Å from H2A and the deepest hole (-0.56 e.Å^-3^) is located 1.47 Å from H2A. The crystal structure was twinned by a pseudo-twofold rotation about (0 1 0) with a refined BASF ratio of 0.115 (1)/0.885 (1).

### Crystal data

**Table:**

C_14_H_13_BrN_2_O_2_S	*Z* = 2
*M**_r_* = 353.23	*F*(000) = 356
Triclinic, *P*1	*D*_x_ = 1.662 Mg m^−^^3^
Hall symbol: -P 1	Mo *K*α radiation, λ = 0.71073 Å
*a* = 5.9565 (3) Å	Cell parameters from 7222 reflections
*b* = 9.4005 (3) Å	θ = 2.5–34.1°
*c* = 12.8020 (6) Å	µ = 3.06 mm^−^^1^
α = 97.153 (2)°	*T* = 100 K
β = 96.350 (2)°	Block, colourless
γ = 92.125 (1)°	0.57 × 0.14 × 0.07 mm
*V* = 705.95 (5) Å^3^	

### Data collection

**Table:**

Bruker SMART APEXII CCD area-detector diffractometer	5040 independent reflections
Radiation source: fine-focus sealed tube	4098 reflections with *I* > 2˘*I*)
graphite	*R*_int_ = 0.034
φ and ω scans	θ_max_ = 32.5°, θ_min_ = 2.2°
Absorption correction: multi-scan (*SADABS*; Bruker, 2005)	*h* = −9→8
*T*_min_ = 0.276, *T*_max_ = 0.819	*k* = −14→14
18030 measured reflections	*l* = −18→19

### Refinement

**Table:**

Refinement on *F*^2^	Primary atom site location: structure-invariant direct methods
Least-squares matrix: full	Secondary atom site location: difference Fourier map
*R*[*F*^2^ > 2σ(*F*^2^)] = 0.040	Hydrogen site location: inferred from neighbouring sites
*wR*(*F*^2^) = 0.106	H atoms treated by a mixture of independent and constrained refinement
*S* = 1.11	*w* = 1/[σ^2^(*F*_o_^2^) + (0.0387*P*)^2^ + 1.0631*P*] where *P* = (*F*_o_^2^ + 2*F*_c_^2^)/3
5040 reflections	(Δ/σ)_max_ < 0.001
187 parameters	Δρ_max_ = 0.63 e Å^−^^3^
0 restraints	Δρ_min_ = −0.56 e Å^−^^3^

### Special details

**Table:**

Experimental. The crystal was placed in the cold stream of an Oxford Cyrosystems Cobra open-flow nitrogen cryostat (Cosier & Glazer, 1986) operating at 100.0 (1) K.
Geometry. All e.s.d.'s (except the e.s.d. in the dihedral angle between two l.s. planes) are estimated using the full covariance matrix. The cell e.s.d.'s are taken into account individually in the estimation of e.s.d.'s in distances, angles and torsion angles; correlations between e.s.d.'s in cell parameters are only used when they are defined by crystal symmetry. An approximate (isotropic) treatment of cell e.s.d.'s is used for estimating e.s.d.'s involving l.s. planes.
Refinement. Refinement of *F*^2^ against ALL reflections. The weighted *R*-factor *wR* and goodness of fit *S* are based on *F*^2^, conventional *R*-factors *R* are based on *F*, with *F* set to zero for negative *F*^2^. The threshold expression of *F*^2^ > σ(*F*^2^) is used only for calculating *R*-factors(gt) *etc*. and is not relevant to the choice of reflections for refinement. *R*-factors based on *F*^2^ are statistically about twice as large as those based on *F*, and *R*- factors based on ALL data will be even larger.

### Fractional atomic coordinates and isotropic or equivalent isotropic displacement parameters (Å^2^)

**Table:**

	*x*	*y*	*z*	*U*_iso_*/*U*_eq_	
Br1	0.81285 (5)	0.24366 (3)	−0.11229 (2)	0.02400 (8)	
S1	0.25512 (10)	0.69735 (6)	0.50609 (4)	0.01269 (11)	
O1	0.1164 (3)	0.66003 (19)	0.58555 (14)	0.0166 (3)	
O2	0.4777 (3)	0.76093 (19)	0.53840 (15)	0.0171 (3)	
N1	0.2703 (4)	0.5426 (2)	0.43423 (17)	0.0147 (4)	
N2	0.3865 (4)	0.5374 (2)	0.34562 (16)	0.0149 (4)	
C1	−0.1178 (4)	0.7666 (3)	0.3846 (2)	0.0162 (4)	
H1A	−0.1895	0.6830	0.4034	0.019*	
C2	−0.2334 (4)	0.8507 (3)	0.3168 (2)	0.0187 (5)	
H2A	−0.3851	0.8231	0.2884	0.022*	
C3	−0.1313 (5)	0.9747 (3)	0.2894 (2)	0.0179 (5)	
C4	0.0912 (5)	1.0145 (3)	0.3328 (2)	0.0194 (5)	
H4A	0.1614	1.1000	0.3162	0.023*	
C5	0.2112 (4)	0.9315 (3)	0.3994 (2)	0.0162 (4)	
H5A	0.3632	0.9587	0.4275	0.019*	
C6	0.1059 (4)	0.8075 (2)	0.42462 (19)	0.0139 (4)	
C7	0.3238 (4)	0.4296 (3)	0.2753 (2)	0.0159 (4)	
H7A	0.1980	0.3695	0.2850	0.019*	
C8	0.4385 (4)	0.3958 (3)	0.18089 (19)	0.0156 (4)	
C9	0.3420 (5)	0.2868 (3)	0.1030 (2)	0.0190 (5)	
H9A	0.2005	0.2417	0.1106	0.023*	
C10	0.4502 (5)	0.2435 (3)	0.0144 (2)	0.0208 (5)	
H10A	0.3833	0.1699	−0.0387	0.025*	
C11	0.6578 (4)	0.3098 (3)	0.0051 (2)	0.0176 (5)	
C12	0.7559 (5)	0.4195 (3)	0.0805 (2)	0.0196 (5)	
H12A	0.8971	0.4646	0.0722	0.023*	
C13	0.6460 (5)	0.4627 (3)	0.1682 (2)	0.0180 (5)	
H13A	0.7119	0.5381	0.2200	0.022*	
C14	−0.2579 (5)	1.0639 (3)	0.2146 (2)	0.0255 (6)	
H14A	−0.2504	1.1643	0.2471	0.038*	
H14B	−0.4164	1.0282	0.2000	0.038*	
H14C	−0.1893	1.0572	0.1481	0.038*	
H1N1	0.160 (6)	0.483 (4)	0.436 (3)	0.020 (8)*	

### Atomic displacement parameters (Å^2^)

**Table:**

	*U*^11^	*U*^22^	*U*^33^	*U*^12^	*U*^13^	*U*^23^
Br1	0.02205 (14)	0.03319 (15)	0.01596 (12)	0.00426 (11)	0.00449 (10)	−0.00286 (10)
S1	0.0128 (3)	0.0121 (2)	0.0130 (2)	−0.00060 (19)	0.0009 (2)	0.00153 (18)
O1	0.0189 (9)	0.0168 (8)	0.0144 (8)	−0.0017 (7)	0.0046 (7)	0.0014 (6)
O2	0.0138 (8)	0.0154 (8)	0.0213 (9)	−0.0018 (6)	−0.0020 (7)	0.0039 (6)
N1	0.0160 (10)	0.0117 (8)	0.0167 (9)	−0.0009 (7)	0.0047 (8)	0.0009 (7)
N2	0.0151 (10)	0.0159 (9)	0.0141 (9)	0.0022 (7)	0.0030 (7)	0.0020 (7)
C1	0.0137 (11)	0.0157 (10)	0.0186 (11)	−0.0015 (8)	0.0005 (9)	0.0017 (8)
C2	0.0155 (11)	0.0206 (11)	0.0190 (11)	0.0027 (9)	−0.0012 (9)	0.0010 (9)
C3	0.0232 (13)	0.0135 (10)	0.0158 (10)	0.0046 (9)	−0.0007 (9)	−0.0011 (8)
C4	0.0237 (13)	0.0123 (10)	0.0219 (12)	0.0007 (9)	0.0014 (10)	0.0027 (9)
C5	0.0166 (11)	0.0138 (10)	0.0174 (11)	−0.0009 (8)	0.0007 (9)	0.0006 (8)
C6	0.0150 (10)	0.0131 (9)	0.0136 (10)	0.0004 (8)	0.0020 (8)	0.0017 (8)
C7	0.0154 (11)	0.0151 (10)	0.0178 (11)	0.0011 (8)	0.0029 (9)	0.0031 (8)
C8	0.0159 (11)	0.0159 (10)	0.0154 (10)	0.0034 (8)	0.0011 (9)	0.0036 (8)
C9	0.0168 (12)	0.0195 (11)	0.0202 (12)	0.0015 (9)	0.0013 (9)	0.0007 (9)
C10	0.0207 (13)	0.0228 (12)	0.0169 (11)	0.0010 (10)	0.0005 (10)	−0.0033 (9)
C11	0.0175 (11)	0.0214 (11)	0.0142 (10)	0.0045 (9)	0.0022 (9)	0.0023 (9)
C12	0.0188 (12)	0.0212 (11)	0.0197 (12)	0.0010 (9)	0.0055 (10)	0.0037 (9)
C13	0.0195 (12)	0.0170 (11)	0.0172 (11)	−0.0014 (9)	0.0028 (9)	0.0008 (9)
C14	0.0335 (16)	0.0178 (11)	0.0226 (13)	0.0073 (11)	−0.0081 (11)	0.0010 (9)

### Geometric parameters (Å, °)

**Table:**

Br1—C11	1.901 (2)	C5—C6	1.393 (3)
S1—O2	1.4297 (19)	C5—H5A	0.9500
S1—O1	1.4469 (18)	C7—C8	1.460 (3)
S1—N1	1.633 (2)	C7—H7A	0.9500
S1—C6	1.756 (2)	C8—C9	1.396 (4)
N1—N2	1.389 (3)	C8—C13	1.400 (4)
N1—H1N1	0.85 (3)	C9—C10	1.390 (4)
N2—C7	1.283 (3)	C9—H9A	0.9500
C1—C2	1.390 (3)	C10—C11	1.388 (4)
C1—C6	1.395 (3)	C10—H10A	0.9500
C1—H1A	0.9500	C11—C12	1.385 (4)
C2—C3	1.396 (4)	C12—C13	1.387 (4)
C2—H2A	0.9500	C12—H12A	0.9500
C3—C4	1.398 (4)	C13—H13A	0.9500
C3—C14	1.509 (4)	C14—H14A	0.9800
C4—C5	1.386 (4)	C14—H14B	0.9800
C4—H4A	0.9500	C14—H14C	0.9800
			
O2—S1—O1	119.47 (11)	N2—C7—C8	122.5 (2)
O2—S1—N1	109.68 (11)	N2—C7—H7A	118.8
O1—S1—N1	102.07 (11)	C8—C7—H7A	118.8
O2—S1—C6	108.68 (11)	C9—C8—C13	119.0 (2)
O1—S1—C6	109.36 (11)	C9—C8—C7	118.2 (2)
N1—S1—C6	106.81 (11)	C13—C8—C7	122.7 (2)
N2—N1—S1	118.59 (16)	C10—C9—C8	120.9 (2)
N2—N1—H1N1	120 (2)	C10—C9—H9A	119.5
S1—N1—H1N1	114 (2)	C8—C9—H9A	119.5
C7—N2—N1	113.5 (2)	C11—C10—C9	118.7 (2)
C2—C1—C6	118.6 (2)	C11—C10—H10A	120.6
C2—C1—H1A	120.7	C9—C10—H10A	120.6
C6—C1—H1A	120.7	C12—C11—C10	121.6 (2)
C1—C2—C3	121.4 (2)	C12—C11—Br1	119.75 (19)
C1—C2—H2A	119.3	C10—C11—Br1	118.67 (19)
C3—C2—H2A	119.3	C11—C12—C13	119.3 (2)
C2—C3—C4	118.5 (2)	C11—C12—H12A	120.4
C2—C3—C14	120.8 (2)	C13—C12—H12A	120.4
C4—C3—C14	120.6 (2)	C12—C13—C8	120.5 (2)
C5—C4—C3	121.2 (2)	C12—C13—H13A	119.8
C5—C4—H4A	119.4	C8—C13—H13A	119.8
C3—C4—H4A	119.4	C3—C14—H14A	109.5
C4—C5—C6	119.1 (2)	C3—C14—H14B	109.5
C4—C5—H5A	120.5	H14A—C14—H14B	109.5
C6—C5—H5A	120.5	C3—C14—H14C	109.5
C5—C6—C1	121.2 (2)	H14A—C14—H14C	109.5
C5—C6—S1	120.30 (19)	H14B—C14—H14C	109.5
C1—C6—S1	118.52 (18)		
			
O2—S1—N1—N2	−54.7 (2)	O2—S1—C6—C1	−179.30 (19)
O1—S1—N1—N2	177.61 (18)	O1—S1—C6—C1	−47.3 (2)
C6—S1—N1—N2	62.9 (2)	N1—S1—C6—C1	62.4 (2)
S1—N1—N2—C7	−156.20 (19)	N1—N2—C7—C8	−173.9 (2)
C6—C1—C2—C3	−0.8 (4)	N2—C7—C8—C9	−172.5 (2)
C1—C2—C3—C4	−0.6 (4)	N2—C7—C8—C13	10.9 (4)
C1—C2—C3—C14	179.2 (2)	C13—C8—C9—C10	0.7 (4)
C2—C3—C4—C5	1.6 (4)	C7—C8—C9—C10	−176.0 (2)
C14—C3—C4—C5	−178.3 (2)	C8—C9—C10—C11	0.6 (4)
C3—C4—C5—C6	−1.0 (4)	C9—C10—C11—C12	−1.5 (4)
C4—C5—C6—C1	−0.5 (4)	C9—C10—C11—Br1	176.8 (2)
C4—C5—C6—S1	178.40 (19)	C10—C11—C12—C13	1.0 (4)
C2—C1—C6—C5	1.4 (4)	Br1—C11—C12—C13	−177.21 (19)
C2—C1—C6—S1	−177.52 (19)	C11—C12—C13—C8	0.3 (4)
O2—S1—C6—C5	1.8 (2)	C9—C8—C13—C12	−1.2 (4)
O1—S1—C6—C5	133.8 (2)	C7—C8—C13—C12	175.4 (2)
N1—S1—C6—C5	−116.5 (2)		

### Hydrogen-bond geometry (Å, °)

**Table:**

*D*—H···*A*	*D*—H	H···*A*	*D*···*A*	*D*—H···*A*
N1—H1N1···O1^i^	0.85 (4)	2.06 (4)	2.902 (3)	171 (4)
C14—H14A···O1^ii^	0.98	2.57	3.420 (3)	145

Symmetry codes: (i) −*x*, −*y*+1, −*z*+1; (ii) −*x*, −*y*+2, −*z*+1.
